# Intrinsic and chemo-sensitizing activity of SMAC-mimetics on high-risk childhood acute lymphoblastic leukemia

**DOI:** 10.1038/cddis.2015.382

**Published:** 2016-01-14

**Authors:** M Schirmer, L Trentin, M Queudeville, F Seyfried, S Demir, E Tausch, S Stilgenbauer, S M Eckhoff, L H Meyer, K-M Debatin

**Affiliations:** 1 Department of Pediatrics and Adolescent Medicine, Ulm University Medical Center, Ulm, Germany; 2 Department of Internal Medicine III, Ulm University Medical Center, Ulm, Germany

## Abstract

SMAC-mimetics represent a targeted therapy approach to overcome apoptosis resistance in many tumors. Here, we investigated the efficacy of the SMAC-mimetic BV6 in B-cell precursor acute lymphoblastic leukemia (BCP-ALL). In ALL cell lines, intrinsic apoptosis sensitivity was associated with rapid cIAP degradation, NF-*κ*B activation, TNF-*α* secretion and induction of an autocrine TNF-*α*-dependent cell death loop. This pattern of responsiveness was also observed upon *ex vivo* analysis of 40 primograft BCP-ALL samples. Treatment with BV6 induced cell death in the majority of ALL primografts including leukemias with high-risk and poor-prognosis features. Inhibition of cell death by the TNF receptor fusion protein etanercept demonstrated that BV6 activity is dependent on TNF-*α*. In a preclinical NOD/SCID/huALL model of high-risk ALL, marked anti-leukemia effectivity and significantly prolonged survival were observed upon BV6 treatment. Interestingly, also *in vivo*, intrinsic SMAC-mimetic activity was mediated by TNF-*α*. Importantly, BV6 increased the effectivity of conventional induction therapy including vincristine, dexamethasone and asparaginase leading to prolonged remission induction. These data suggest SMAC-mimetics as an important addendum to efficient therapy of pediatric BCP-ALL.

In childhood acute lymphoblastic leukemia (ALL), major improvements in therapy and supportive care have led to increased survival rates.^1^ In current treatment protocols, patients are assigned to multi-agent chemotherapy regimens of different intensities based on the patient's individual risk profile and probability to encounter relapse.^2^ However, 20% of patients relapse or do not respond well to therapy.^3, 4^ Therefore, new treatment strategies are required to overcome resistance mechanisms and enhance outcome. Defects in cell death mechanisms contribute to resistance and treatment failure in many cancers, including leukemia.^5, 6^ We previously described, that deficient apoptosis signaling in primary, patient-derived leukemia cells is indicative for early patient relapse and associated with rapid engraftment in NOD/SCID mice.^7, 8, 9, 10^

Two major apoptosis signaling pathways are known, the death receptor (extrinsic) and mitochondrial (intrinsic) pathway.^11^ Within the extrinsic pathway, activation of death receptors of the tumor necrosis factor (TNF) superfamily activate caspase-8 via a death-inducing signaling complex (DISC) leading to activation of effector caspases.^12^ Activation of intrinsic signaling triggers mitochondrial release of cytochrome *c* and SMAC (second mitochondria-derived activator of caspase). Released cytochrome *c* forms a complex together with caspase-9, the apoptosome, which cleaves and activates caspase-3. SMAC antagonizes inhibitor of apoptosis proteins (IAPs), thus enabling apoptosis signaling.^13^

IAPs constitute a family of structurally related proteins, defined by the presence of at least one baculoviral IAP repeat (BIR) domain, which can bind to active subunits of caspases, thereby inhibiting their function. Some IAPs (e.g., cIAP) also possess a RING domain conferring E3 ligase activity mediating ubiquitination reactions.^14^

Since IAPs are often overexpressed and associated with inferior outcome in different malignancies including childhood acute leukemias, they may serve as an attractive target for therapeutic intervention.^15, 16, 17, 18^ Previously, IAP antagonists, binding to selected BIR domains of IAPs and leading to cell death sensitization were designed.^16, 19, 20, 21^ Recently, a new class of molecules mimicking the N-terminal domain of SMAC (SMAC-mimetics) was developed, which bivalently bind to both BIR2 and BIR3 domains, and possess additional intrinsic apoptogenic activity via a TNF-*α* feed forward loop.^22, 23^ Within this TNF-*α* loop, SMAC-mimetics stimulate the E3-ubiquitine ligase activity of cIAPs, leading to autoubiquitination and subsequent proteasomal degradation of cIAPs. Depletion of cIAPs enables accumulation of NF-*κ*B-inducing kinase (NIK), resulting in non-canonical activation of NF-*κ*B and NF-*κ*B target gene expression, including *TNFA*, which stimulates TNFR1 in an autocrine loop.^23^ In the absence of cIAP, TNFR1-stimulation triggers the assembly of the secondary RIP1-containing cytoplasmatic complex (complex II), leading to caspase-8 activation and cell death induction.^24^ SMAC-mimetics may induce cell death dependently or independently of TNF-*α*,^25, 26, 27^ and we previously showed, that counteracting caspase inhibition by IAP antagonists sensitizes cancer cells for death receptor ligand- and chemotherapy-induced apoptosis.^28, 29^

Here, we analyze the therapeutic potential of the SMAC-mimetic BV6 in pediatric B-cell precursor (BCP)-ALL *in vitro*, *ex vivo* and in a preclinical model *in vivo*, and define the molecular requirements for intrinsic activity.

## Results

### Heterogeneous BV6 sensitivity of BCP-ALL cell lines

**Figure 1:**
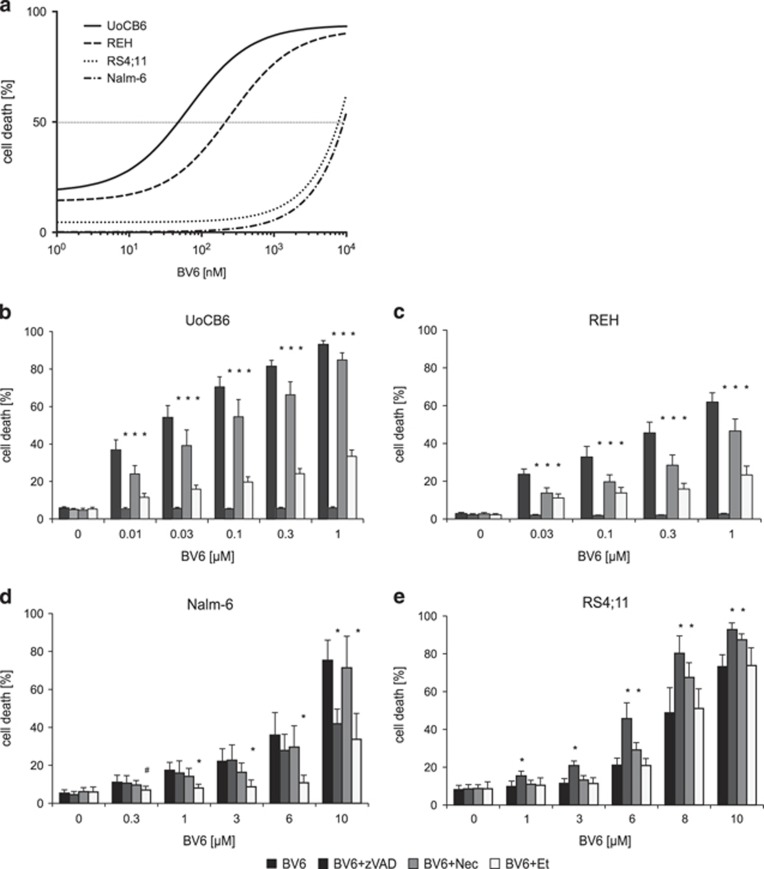
Intrinsic activity of BV6 on BCP-ALL cell lines. (**a**) Heterogeneous sensitivity of BCP-ALL cell lines for cell death after exposure to BV6 (48 h). UoCB6 and REH display half-maximal inhibitory concentration (IC_50_) values at nanomolar concentrations in contrast to Nalm-6 and RS4;11 showing sensitivity in the micromolar range. (**b**–**e**) Inhibition of BV6-induced cell death (48-h exposure to BV6 at indicated concentrations) by 20 *μ*M zVAD.fmk (zVAD), 30 *μ*M Necrostatin-1 (Nec-1), or 40 *μ*g/ml etanercept (Et). Percentages of dead cells were estimated by flow cytometry according to forward and side scatter criteria, three independent experiments each performed in triplicate, estimation of IC_50_ (**a**), comparison of BV6 to BV6 and the respective inhibitors; mean and S.D. are indicated; significance by Mann-Whitney *U*-test; *P<0.01 and ^#^*P*<0.05 (**b**–**e**)

We assessed cell death induction upon exposure of the SMAC-mimetic BV6 in four BCP-ALL cell lines (UoCB6, REH, Nalm-6 and RS4;11). In two of the cell lines, BV6 induced cell death at nanomolar concentrations (half-maximal inhibitory concentration, IC_50_ UoCB6: 66.1 nM; IC_50_ REH: 251.1 nM). In contrast, Nalm-6 and RS4;11 were resistant at concentrations in this nanomolar range beginning to show sensitivity for BV6 at clearly higher micromolar concentrations (Figure 1a). Nonmalignant lymphocytes (PBLs) obtained from healthy donors were largely refractory to BV6-induced cell death at nanomolar up to micromolar concentrations, demonstrating that BV6 is non-toxic to normal PBLs at concentrations sufficient to induce apoptosis in BV6-sensitive leukemia cells (Supplementary Figure 1A).

To address the mode of BV6-induced cell death, BCP-ALL cell lines were pretreated with the pan-caspase-inhibitor zVAD.fmk and the necrosis-inhibitor Necrostatin-1 (Nec-1). BV6-induced cell death was completely blocked by the pan-caspase-inhibitor zVAD.fmk in sensitive cell lines indicating apoptosis as the primary cell death mechanism in these cells (Figure 1b and c). In contrast, cell death induced by high concentrations of BV6 in Nalm-6 cells was only reduced to a minor extent by zVAD.fmk (Figure 1d), and zVAD.fmk induced even more cell death in RS4;11 cells (Figure 1e). Nec-1 had no inhibitory effect on resistant cell lines and only a minimal inhibitory effect on sensitive cell lines (Figure 1b and e), also upon additional incubation with zVAD.fmk (Supplementary Figure 1B and C), indicating the absence of necrotic or necroptotic cell death.

SMAC-mimetics have been reported to induce cell death via a TNF-*α* loop. Interestingly, the TNFR fusion protein etanercept inhibited BV6-induced cell death in sensitive cell lines and to some extent in Nalm-6 but not in RS4;11 cells, suggesting involvement of TNF-*α* in cells sensitive to BV6-mediated apoptosis (Figure 1b to e).

Thus, we observed heterogeneous sensitivities toward the SMAC-mimetic BV6, which effectively induces TNF-*α*-dependent apoptosis at nanomolar concentrations in sensitive BCP-ALL cell lines, but importantly not in non-malignant, healthy lymphocytes.

### BV6 induces an autocrine TNF-*α* loop in BCP-ALL cell lines

**Figure 2:**
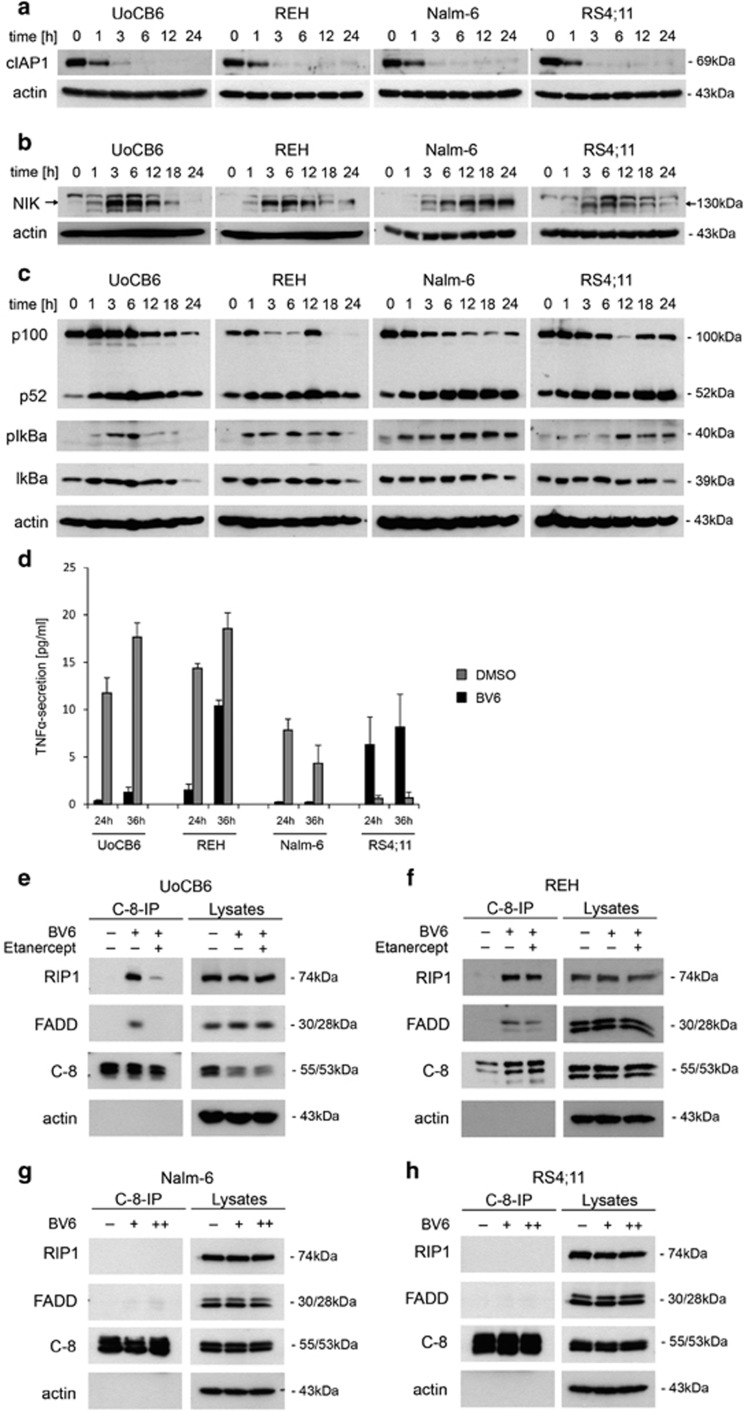
TNF-*α* signaling upon BV6 exposure. Western blot analysis of (**a**) cIAP1 degradation, (**b**) NIK accumulation and (**c**) NF-*κ*B activation in BCP-ALL cell lines exposed to BV6 for indicated time points (UoCB6 and REH: 1 *μ*M BV6; Nalm-6 and RS4;11: 10 *μ*M BV6; or equivalent doses of DMSO for 24 h). For each analysis, one representative blot of three is shown. Arrows indicate NIK. (**d**) Increased TNF-*α* protein levels in cellular supernatants of BCP-ALL cell lines treated with BV6 or control as indicated (mean and S.D. of three independent experiments each performed in duplicate). (**e**–**h**) TNFR complex II formation upon BV6 exposure (UoCB6 and REH: 1 *μ*M; Nalm-6 and RS4;11: 1 *μ*M (+) or 10 *μ*M (++) BV6) and dependency on TNF-*α* (with or without 40 *μ*g/ml etanercept). Caspase-8 immunoprecipitation in the presence of 10 *μ*M zVAD.fmk (UoCB6, REH and Nalm-6), and western blot analysis of indicated proteins (one representative experiment of three is shown)

Next, we investigated involvement of TNF-*α* in molecular detail. SMAC-mimetics have been reported to trigger autoubiquitination and proteasomal degradation of cIAPs, NIK accumulation and NF-*κ*B activation followed by TNF-*α* production and secretion. Interestingly, BV6 caused rapid cIAP1 degradation in all cell lines (Figure 2a; Supplementary Figure 3). However, in contrast to cIAP1, cIAP2 is hardly detectable in BCP-ALL cell lines and was not affected by BV6 (Supplementary Figure 2A and B). Subsequent to cIAP1 degradation, we observed increasing NIK levels, indicating an accumulation of NIK (Figure 2b; Supplementary Figure 3A and B). In line, a decrease in p100 and an increase in p52 were observed, pointing to activation of the non-canonical NF-*κ*B pathway. Moreover, increasing pIkB levels indicated activation of the canonical NF-*κ*B pathway (Figure 2c; Supplementary Figure 3C). Interestingly, TNF-*α* secretion was induced in BV6-sensitive cell lines, but to a lesser extend or not in BV6-insensitive cells (Figure 2d).

### BV6 activates apoptosis signaling pathways in a TNF-*α*-dependent manner, mediated by the TNFR complex II

In order to analyze whether the TNFR complex II is required for BV6-mediated cell death induction, complex formation (caspase-8, RIP1 and FADD) was investigated.

Importantly, caspase-8 immunoprecipitation revealed marked increase of the TNFR complex II in BV6-sensitive cells (Figure 2e and h; Supplementary Figure 3D and G). Addition of etanercept attenuated this complex formation, consistent with the requirement of secreted TNF-*α* in this model of apoptosis (Figure 2e and f).

**Figure 3:**
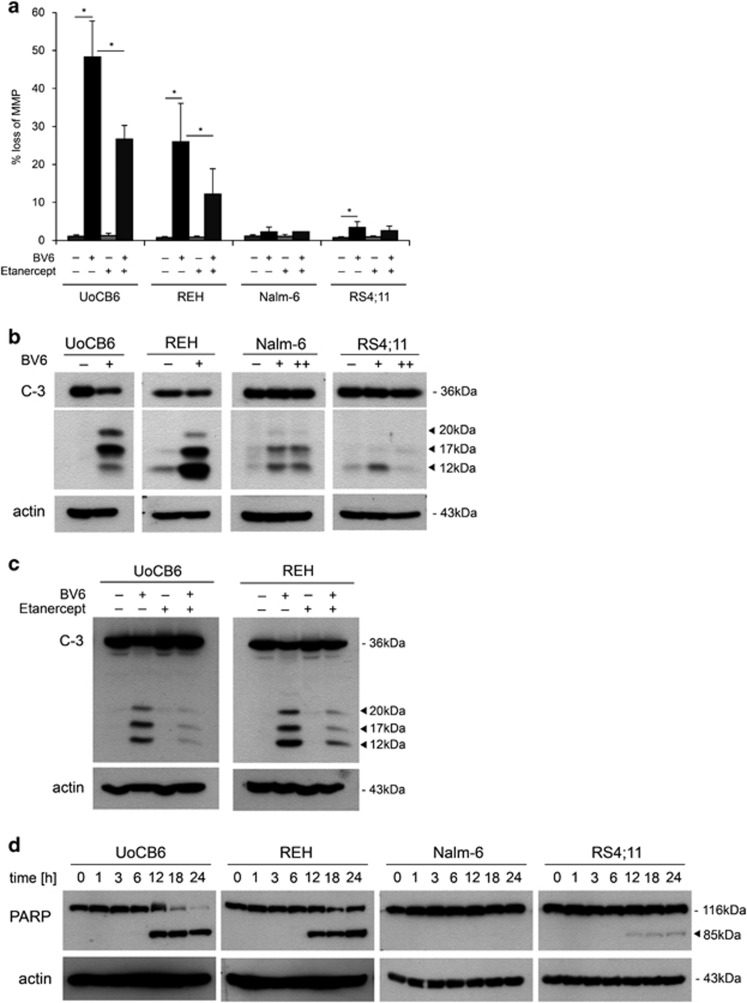
Apoptosis signaling upon BV6 treatment. (**a**) Loss of mitochondrial membrane potential (MMP) in BCP-ALL cell lines upon BV6 treatment (UoCB6 and REH: 1*μ*M; Nalm-6 and RS4;11: 10 *μ*M BV6; 24 h, with or without 40 *μ*g/ml etanercept; mean and S.D. of three independent experiments each performed in triplicate, significance by Mann-Whitney *U*-test, *P<0.01. (**b**) Caspase-3 activation in BCP-ALL cell lines upon BV6 treatment (UoCB6 and REH: 1 *μ*M; Nalm-6 and RS4;11: 1 *μ*M (+) or 10 *μ*M (++) BV6; 24 h), and (**c**) TNF-*α* dependency of the BV6-sensitive leukemia cell lines UoCB6 and REH (additional treatment with or without 40 *μ*g/ml etanercept), western blot analysis. (**d**) PARP cleavage upon BV6 treatment for indicated time points (UoCB6 and REH: 1 *μ*M; Nalm-6 and RS4;11: 10 *μ*M BV6), western blot analysis. For each western blot analysis, one representative experiment out of three is displayed

Next, we evaluated cell death signaling downstream of the TNFR complex II. In contrast to resistant cells, a significant loss of mitochondrial membrane potential, a constant feature of type-II cells and largely observed in ALL, was detected upon BV6 in sensitive ALL cell lines, which was clearly reduced by etanercept, indicating activation of the intrinsic apoptosis pathway downstream of TNFR complex II (Figure 3a). Caspase-3 activation with decrease in proforms and increase in cleavage products was predominantly seen in the BV6-sensitive cell lines (Figure 3b; Supplementary Figure 4A) and almost completely inhibited by etanercept (Figure 3c; Supplementary Figure 4B), indicating TNF-*α*-dependent caspase activation downstream of the TNFR complex II. Consistently, PARP was cleaved in sensitive but not in resistant cell lines (Figure 3d; Supplementary Figure 4C). These findings highlight the essential requirement of the TNFR complex for BV6-induced activation of cell death signaling.

### Apoptosis induction by BV6 is dependent on RIP1

**Figure 4:**
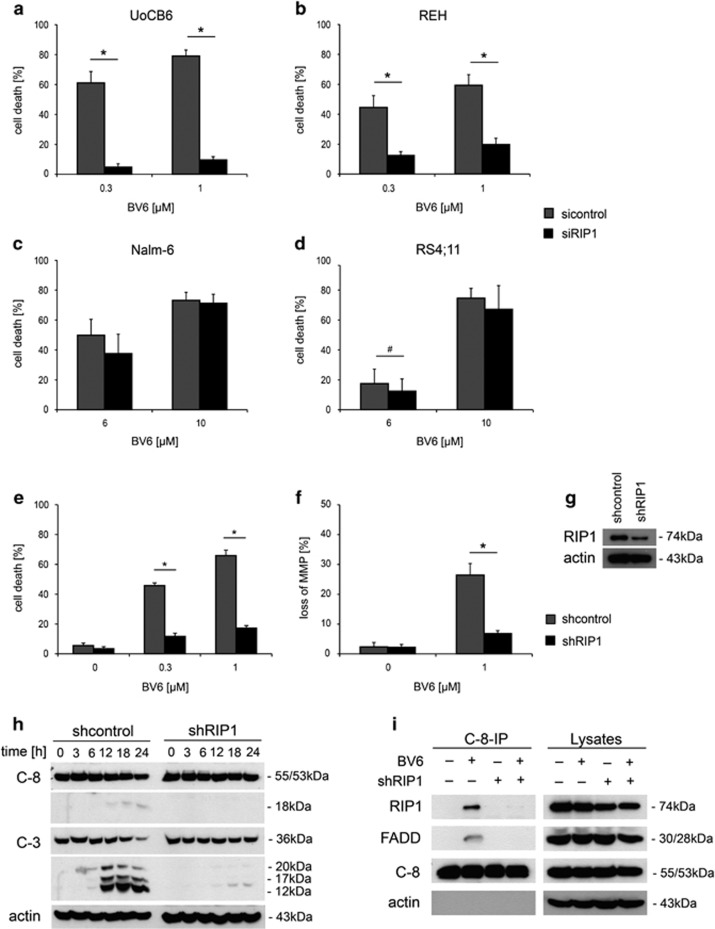
Dependency of BV6-induced cell death on RIP1. Block of BV6-induced cell death (exposure at indicated concentrations for 48 h) by RIP1 knockdown in BV6-sensitive (**a** and **b**) but not BV6-insensitive (**c** and **d**) cell lines. Mean and S.D. of three independent experiments each performed in triplicate are shown, significance by Mann-Whitney *U*-test; *P<0.01 and ^#^*P*<0.05. (**e**) Blocked BV6-induced cell death (48-h exposure at indicated concentrations), and (**f**) loss of mitochondrial membrane potential (MMP; 24 h, 1 *μ*M BV6) in REH cells with stable shRNA-mediated RIP1 knockdown. Mean and S.D. of three independent experiments each performed in triplicate are shown, significance by Mann-Whitney *U*-test; *P<0.01. (**g**) Efficient knockdown of RIP1 in shRNA *versus* control-transduced REH cells is shown by western blot analysis. (**h**) Absence of caspase-8 and -3 activation (1 *μ*M BV6 for indicated time points) in REH cells upon shRNA-mediated RIP1 knockdown, as well as (**i**) impaired complex II formation (1 *μ*M BV6 for 18 h, caspase-8 immunoprecipitation in the presence of 10 *μ*M zVAD.fmk and western blot analysis of indicated proteins)

cIAPs have been reported to modulate RIP1 by ubiquitination with their E3 ligase activity, thereby determining the function of RIP1 in the TNFR complex. Therefore, we explored the role of RIP1 in TNFR complex II-mediated apoptosis induction upon BV6 treatment. RNA interference-mediated knockdown of RIP1 reduced SMAC-mimetic-induced cell death clearly in BV6-sensitive cell lines (Figure 4a and b), whereas this effect was not observed in BV6-insensitive cell lines (Figure 4c and d). Reduced RIP1 protein expression upon siRNA-mediated knockdown was confirmed by western blot analyses (Supplementary Figure 5A and D).

To further elucidate the role of RIP1, we analyzed downstream apoptosis signaling in the presence or absence of RIP1 in the BV6-sensitive cell line REH. REH cells with stable shRNA-mediated RIP1 knockdown showed a clear reduction of cell death (Figure 4e), blocked activation of intrinsic and extrinsic apoptosis signaling as indicated by absence of loss of mitochondrial membrane potential (Figure 4f) and lack of caspase activation (Figure 4h; Supplementary Figure 5E). Moreover, caspase-8 immunoprecipitation showed a profound reduction of FADD recruitment in the absence of RIP1 (Figure 4i; Supplementary Figure 5F), demonstrating that RIP1 is required for complex II formation.

Taken together, these data show that apoptosis induction by the SMAC-mimetic BV6 in BCP-ALL is dependent on RIP1.

### BV6 induces cell death in primograft leukemia samples dependent on TNF-*α* including high-risk and poor outcome ALL

**Figure 5:**
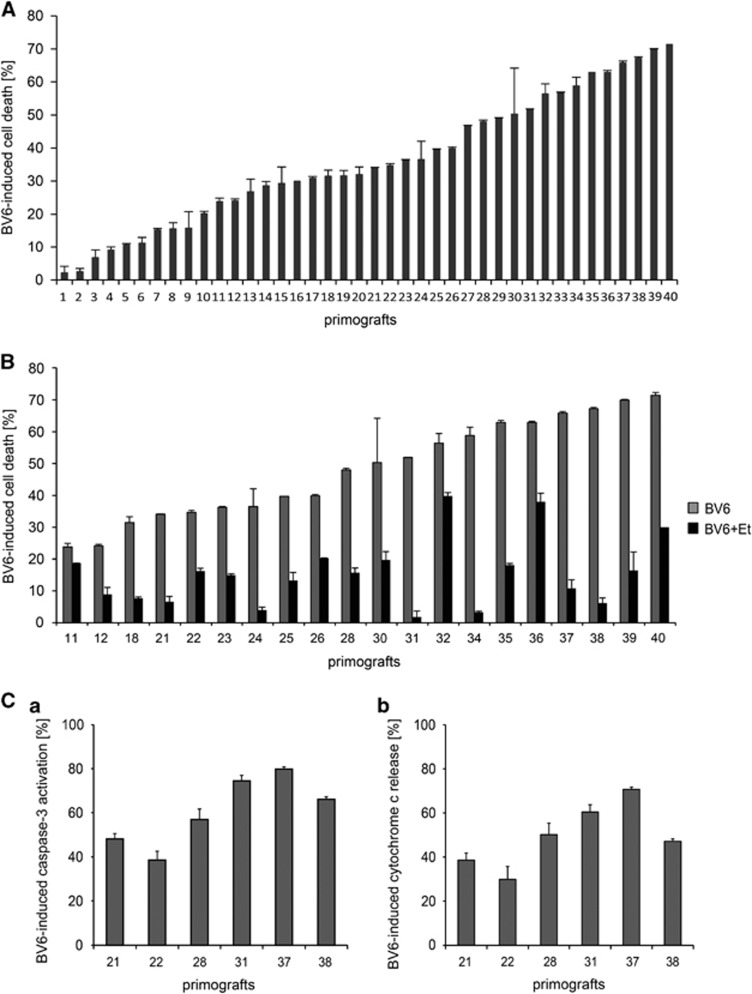
BV6-induced cell death in primary ALL. Cell death induction in primary, patient-derived BCP-ALL samples (*n*=40) by BV6 (**A**) and dependency on TNF-*α* (*n*=20 primografts; **B**). Cell death by flow cytometry according to forward and side scatter criteria upon treatment with 1 *μ*M BV6 for 48 h and with 40 *μ*g/ml (BV6+Et) or without, BV6-induced cell death was calculated as the difference of total and spontaneous cell death; experiments performed in triplicate; mean and S.D. are indicated. (**C**) Activation of caspase-3 (a), and cytochrome *c* release (b) upon BV6 treatment (1 *μ*M, 18 h) in primograft samples. Percentages of cells with activated caspase-3 and cells with released cytochrome *c* were estimated by flow cytometry in triplicate, bars represent mean values and S.D.

Next, we investigated the effects of BV6 in a series of 40 primograft ALL samples established in our NOD/SCID/huALL mouse model by xenografting therapy-naive patient ALL cells obtained at first diagnosis or relapse of pediatric BCP-ALL patients (Table 1). Cell death induction was observed in the majority of leukemias with two-thirds of primografts showing induction of 25% or more cell death upon exposure to BV6 (Figure 5A).

In a subset of 20 samples, etanercept inhibited BV6-mediated cell death indicating TNF-*α* dependency also in patient-derived ALL primografts as observed in sensitive cell lines (Figure 5B).

However, no association of leukemia or patient characteristics and clinical outcome with BV6 sensitivity was observed. Interestingly, leukemia samples derived from BFM-high-risk stratified patients (#11, #12, #21, #23 and #27) or from patients at relapse (#4, #35 and #39) were sensitive to BV6. Previously, we observed that rapid engraftment of patient-derived ALL cells transplanted onto NOD/SCID mice (short time to leukemia, TTL^short^) indicates early patient relapse.^8^ Of note, BV6 sensitivity was also seen in samples of this poor-prognosis phenotype (#6, #8, #11, #22, #23, #24, #27, #28, #31, #33 and #34). Moreover, we described earlier that deficient spontaneous apoptosis signaling in patient and xenograft BCP-ALL cells is associated with poor patient outcome.^7, 9^ By the same strategy, 5 of 16 samples investigated showed intrinsic resistance to spontaneous apoptosis (#6, #11, #22, #31 and #38), of which four were BV6-sensitive. Furthermore, addressing key downstream apoptosis signaling events, we identified mitochondrial cytochrome *c* release and activation of caspase-3, indicating BV6-induced activation of apoptosis signaling in primary BCP-ALL (Figure 5Ca and b).

In summary, SMAC-mimetics induce clear activity in the majority of patient-derived ALL samples, and importantly in leukemias associated with high-risk characteristics, poor patient outcome, relapse and apoptosis deficiency.

### BV6 treatment is highly effective in a preclinical NOD/SCID/huALL mouse model *in vivo*

**Figure 6:**
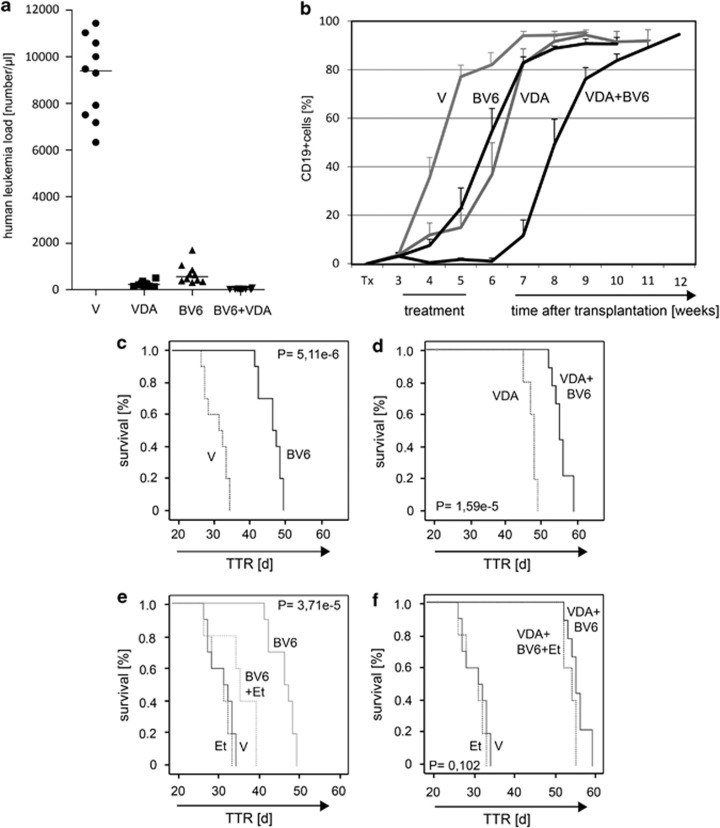
Preclinical activity of BV6 on pediatric high-risk ALL. NOD/SCID mice transplanted with a high-risk BCP-ALL showing presence of human ALL cells in the peripheral blood were treated as indicated. (**a**) Leukemia load (numbers of human leukemia cells in the recipient's peripheral blood at the end of treatment, recipients individual and median values are shown). (**b**) Percentages of human (CD19 positive) ALL cells in the recipient's peripheral blood over time (data points represent mean values and S.D. per group). Superior leukemia-free survival of ALL-bearing animals after treatment with BV6 (**c**), a combination of VDA induction-chemotherapy and BV6 compared to chemotherapy alone (**d**), and *in vivo* TNF-*α* dependency of intrinsic (**e**) but not of chemo-sensitizing BV6 activity (**f**). Probabilities of leukemia-free survival after treatment with vehicle (V), BV6, induction-chemotherapy (vincristine, dexamethasone and asparaginase; VDA) or combinations thereof (10 animals per group) with or without etanercept (Et; five animals per group); TTR, time to leukemia reoccurrence; Kaplan-Meier analysis, log-rank test; *P*, significance

On the basis of these results, we further evaluated the efficacy of BV6 in a preclinical *in vivo* setting on a primograft leukemia sample with rapid NOD/SCID engraftment, and deficient apoptosis signaling derived from a patient who later on suffered from early relapse (#31). Recipient animals were transplanted, and upon leukemia manifestation treated with either BV6 or solvent (10 recipients per group) for 2 weeks. The medication was well tolerated and no signs of *in vivo* toxicity were observed. Interestingly, a significant reduction of leukemia load at the end of therapy, a significant delay of disease reoccurrence and prolonged leukemia-free survival were observed upon BV6 *in vivo* treatment as compared to control-treated animals (Figure 6a to c).

In a clinical setting, however, high-risk disease is unlikely to be treated by one compound alone. Therefore, we combined BV6 with multidrug chemotherapy resembling ALL induction treatment and observed an almost complete reduction of tumor load, a significant delay of leukemia reoccurrence and prolonged survival (Figure 6a, b and d) of animals treated with the combination of SMAC-mimetic and chemotherapy compared to chemotherapy alone.

Importantly, additional administration of etanercept reversed the *in vivo* effect of BV6 single treatment on leukemia-free survival, indicating TNF-*α* dependency also *in vivo* (Figure 6e). Etanercept on its own, however, did not affect leukemia-free survival (Figure 6e). In contrast, abrogation of TNF-*α* signaling had only a minor effect on survival after combination therapy with VDA and BV6, suggesting that the sensitizing effect of BV6 for chemotherapy is only partially TNF-*α* dependent (Figure 6f).

## Discussion

IAPs have been shown to be overexpressed in several cancers including acute leukemia, and to be associated with treatment failure and inferior prognosis.^17, 18^ In line with the prognostic role of intact cell death pathways, we found that deficient apoptosis signaling in primary BCP-ALL cells is indicative of early patient relapse.^7, 8, 9, 10^ Thus, antagonizing IAP proteins by, for example, SMAC-mimetics may represent an attractive strategy to activate the apoptotic machinery and to overcome resistance. In previous studies, we and others have shown that IAP inhibition sensitizes various tumor cells, including acute leukemia cells, for cell death-inducing stimuli *in vitro* and *in vivo*.^28, 29^ Also, single agent activities of IAP inhibitors and small-molecule SMAC-mimetics have been reported for several tumor cell lines, and some primary tumor samples including AML and CLL.^30, 31^ However, the spectrum of activity of SMAC-mimetics in ALL has not been examined in detail so far.

Here we demonstrate, that the small-molecule SMAC-mimetic BV6 is highly effective to induce cell death in ALL cell lines and, most importantly, in a wide range of patient-derived primograft samples, and is effective on high-risk leukemia leading to prolonged survival in a preclinical NOD/SCID/huALL primograft model *in vivo*.

Interestingly, we detected a concentration-dependent sensitivity to BV6 in BCP-ALL cell lines. Cell lines sensitive for low-dose BV6 (UoCB6 and REH) exhibited a full activation of the TNF-*α* feed forward loop: rapid degradation of cIAP, NIK stabilization, NF-*κ*B activation and TNF-*α* secretion, followed by TNF-*α*-dependent apoptosis induction, as demonstrated by addition of the soluble TNFR etanercept, which prevented mitochondrial perturbation, caspase activation and apoptosis. Furthermore, we found that TNF-*α* is secreted by ALL cells and subsequently triggers the formation of the TNFR complex II consisting of caspase-8, RIP1 and FADD. This complex was described to be formed as a result of cIAP degradation by SMAC-mimetics at the stimulated TNFR1 in the presence of de-ubiquitinated RIP1.^24, 32^ In line with this, we found formation of TNFR complex II upon cIAP1 degradation and that complex formation is dependent on TNF-*α*, since the addition of etanercept reduced complex formation. Accordingly, exposure of the BCP-ALL cell line REH to etanercept was reported to decrease BV6-mediated cell death; however, this effect was not observed in the T-ALL line Jurkat.^33^ Furthermore, we provide evidence that the presence of RIP1 is crucial for apoptosis induction upon BV6 treatment. Thus, knockdown of RIP1 abrogated the formation of complex II and profoundly reduced SMAC-mimetic-induced mitochondrial perturbation, caspase activation and apoptosis induction. However, inhibition of the RIP1 kinase by Nec-1 had only a slight effect on apoptosis induction, demonstrating that cell death induction by SMAC-mimetics is dependent on the availability of RIP1 in the complex but not on the kinase activity of RIP1. Although cell lines resistant to BV6 (Nalm-6 and RS4;11) showed rapid cIAP degradation, NIK stabilization and NF-*κ*B activation after BV6 treatment, lower or no enhanced TNF-*α* production was detectable in these cells. The lack of BV6-induced TNF production suggests that sensitivity to the intrinsic activity of SMAC-mimetics at lower concentrations strictly depends on the TNF-*α* loop. However, no associations of BV6-sensitivity and genetic aberrations including *TP53* mutations were observed.

SMAC-mimetics have been reported to have low *ex vivo* activities on different cancer xenografts with a majority of tumors being resistant and a minority showing sensitivity to SMAC-mimetics mostly at higher micromolar concentrations.^34, 35^ In AML and CLL, cells have been reported to be sensitive for SMAC-mimetics in single and combination treatment regimens *ex vivo*, predominantly also at micromolar concentrations.^30, 31^ However, at these higher concentrations toxic effects on monocytes and dendritic cells have been observed.^36^ Recently, SMAC-mimetics have been shown to moderately sensitize primary ALL cells for dexamethasone *ex vivo* and in a non-systemic, subcutaneous leukemia model *in vivo*,^33^ and no *in vivo* intrinsic activity on ALL was observed for the SMAC-mimetic LCL-161.^35^

In contrast, we found that the majority of patient-derived ALL samples is sensitive for the SMAC-mimetic BV6, including leukemias with high-risk/poor prognostic features. Primograft samples, which were sensitive for BV6 showed TNF-*α* dependent cell death induction. Most importantly, we also found that BV6 treatment leads to activation of intrinsic and extrinsic apoptosis pathways, also in leukemias with deficient constitutive apoptosis signaling indicative for early patient relapse. Thus, SMAC-mimetics are effective in leukemias including ALL with high-risk characteristics and intrinsic apoptosis resistance.

Our study also provides clear evidence that the SMAC-mimetic BV6 is highly effective in a BCP-ALL primograft model *in vivo*. Single treatment with BV6 led to a significant reduction of tumor burden and prolonged leukemia-free survival. This intrinsic activity of BV6 was clearly dependent on TNF-*α*, since concomitant *in vivo* treatment with etanercept reversed the effect. The combination of BV6 with conventional chemotherapy was even more effective, resulting in substantial reduction of tumor load and significant delay of post-treatment leukemia reoccurrence. This effect, however, was largely independent of TNF-*α* suggesting that the sensitizing effect of SMAC-mimetics might be mainly due to the prevention of caspase inhibition.

In summary, SMAC-mimetics exhibit high-intrinsic activity in ALL cell lines and in the vast majority of primary ALL samples in a TNF-*α*-dependent manner. BV6 is also highly effective on patient-derived leukemia samples, associated with high-risk/poor-prognosis features leading to significantly prolonged relapse-free survival in a high-risk preclinical NOD/SCID/huALL primograft model, both as single treatment and in combination with conventional chemotherapy. With this, our findings provide important evidence for the potential use of SMAC-mimetics in treatment strategies of childhood BCP-ALL. As SMAC-mimetics recently entered phase II clinical trials as single agents and in combination with chemotherapeutics, they may be deployable for clinical use shortly. Our studies on normal lymphocytes did not reveal any effect at concentrations sufficient to induce cell death in ALL cells, and most SMAC-mimetics have been reported to be well tolerated in phase I and II studies.^37, 38^ Since SMAC-mimetics induce cell death and reactivate apoptosis signaling even in apoptosis-resistant cells of high-risk patients, they may be used in treatment protocols for high-risk/poor-prognosis ALL patients. Furthermore, the increased effect of SMAC-mimetics along with conventional chemotherapeutics offers the opportunity to use them also in standard-risk groups to reduce the dose of cytotoxic drugs, thereby decreasing chemotherapy-related side effects. Our data also define the requirements for the intrinsic activity of BV6 and possibly also other SMAC-mimetics: induction of TNF-*α* and availability of RIP1 in the TNFR complex. These may be used as biomarkers to select ALL patients for treatment with SMAC-mimetics such as BV6. Interestingly, in contrast to the TNF-*α*-dependent intrinsic activity of BV6, the chemotherapy-sensitizing effect appears to be mediated by the ‘classical' SMAC function, that is, inhibition of IAPs. Thus, the chemo-sensitizing effect may be used in IAP-expressing leukemias.

In conclusion, SMAC-mimetics present a promising new therapeutic approach for pediatric ALL, which deserves further investigation in clinical trials.

## Material and Methods

### Cells

Cell lines were obtained from DSMZ, Braunschweig, Germany; UoCB6 cells were kindly provided by Dr. Rowley, Chicago, USA. Cells were mycoplasma-negative, and authenticated by single-tandem-repeat profiling and kept under standard conditions.^39^ All four cell lines are of a BCP immunophenotype (BCP-ALL), REH and UoCB6 were derived from *ETV6*/*RUNX1*^−^-rearranged, RS4;11 from *MLL*/*AF4*-rearranged leukemias.^40^ Analysis of the cell lines for *TP53* mutations by denaturing high-performance liquid chromatography followed by Sanger sequencing of exons 4–10^41^ identified *TP53* mutations in the cell lines REH and RS4;11 while UoCB6 and Nalm-6 were found to be *TP53* wild type.

PBLs were isolated from healthy donors and cultured (37 °C, 5% CO_2_) in RPMI1640 with 10% fetal calf serum (Conco, Wiesbaden, Germany), 10 mM HEPES, 2 mmol/l l-glutamine, 30 units (U)/ml huIL-2 (Biochrom, Berlin, Germany), 100 U/ml penicillin and 100 *μ*g/ml streptomycin (Life Technologies, Frankfurt, Germany). Adhesed monocytes were removed, and lymphocytes were selected according to forward side scatter properties.

### NOD/SCID/huALL

Primograft leukemias were established transplanting patient ALL cells onto female, 6-week-old NOD/SCID mice (NOD/LtSz-scid/scid, Charles River, Germany) as previously described.^7, 8, 39^ Patient samples were obtained after informed consent of patients and/or their legal guardians in accordance with the institution's ethical review board, and all animal experiments were approved by the appropriate authority (Regierungspräsidium Tübingen, TVA-Nr. 1147). Upon leukemia onset, xenograft cells (minimum 90% huCD19-positive cells) were isolated from spleens and used for *ex vivo* analyses or transplanted onto subsequent recipients. All together 40 available patient-derived BCP-ALL samples (obtained after in median 2.5 passages) have been included into this study. Patient and leukemia characteristics are summarized in Table 1. For *in vivo* treatment, ALL cells of primograft #31 were re-transplanted. On the basis of survival times (log-rank test), sample size estimates had been calculated (Ulm University, Institute of Epidemiology and Medical Biometry), and recipients were subdivided into groups of ten by consecutive numbering. Upon appearance of more than 5% human leukemia cells in peripheral blood, vehicle (DMSO); VDA (vincristine, 0.075 mg/kg, once per week; dexamethasone, 2.5 mg/kg, 5 days per week; asparaginase, 500 IU/kg, 5 days per week); BV6 (10 mg/kg, twice per week); or combinations were applied by intraperitoneal (i.p.) injections (investigator is non-blinded) for 2 weeks. In addition, etanercept (10 mg/kg, i.p., twice per week, 2 weeks) or combinations with VDA/BV6 were applied (five mice per group). Human leukemia was monitored weekly in PB by flow cytometry staining for huCD19 (anti-CD19-Allophycocyanin, #555415 and LSR-II cytometer, BD Bioscience, Heidelberg, Germany). White blood cell counts were performed once after completion of treatment. At onset of leukemia-related morbidity, mice were killed, and high leukemia infiltration was confirmed in bone marrow, spleen and PB by flow cytometry as previously described.^8^

### RIP1 knockdown

Experiments were carried out 48 h after transient transfection (Amaxa Nucleofector, Lonza, Basel, Switzerland) with siRNA oligonucleotides (ON-TARGETplus Human RIP1 siRNA SMART pool and NON-TARGETING pool; Dharmacon, Lafayette, USA). REH cells with stable (shRNA) RIP1 knockdown were kindly provided by S Löder and Dr. Fulda, Ulm, Germany.^29^

### Reagents, analysis of cell death signaling

Genentech (San Francisco, USA) kindly provided BV6, Pfizer (New York, USA) provided etanercept, Medac (Hamburg, Germany) provided *E. coli* asparaginase and the Pharmacy, University Medical Center Ulm provided dexamethasone and vincristine. *N*-Benzyloxycarbonyl-Val-Ala-Asp-fluoromethylketone (z.VAD.fmk) was obtained from Bachem (Heidelberg, Germany), Nec-1 from Biomol (Hamburg, Germany) and all other chemicals from Sigma-Aldrich. Western blot analysis was performed as previously described,^42^ using mouse anti-caspase-8 (#ALX-804-242-C100; Alexis Life Sciences, New York, USA); mouse anti-caspase-2 (#611022), rabbit anti-caspase-9 (#556585), mouse anti-RIP (#551041), mouse anti-FADD (#610400; BD Biosciences), rabbit anti-caspase-3 (#9662), rabbit anti-NIK (#4994), mouse anti-pIkBa (#9246), rabbit anti-IkBa (#9242; Cell Signaling, Beverly, USA), rabbit anti-caspase-8 (#1006-1), rabbit anti-cIAP2 (#ab32059; Epitomics, Burlingame, USA), goat anti-cIAP1 (#AF8181; R&D Systems, Wiesbaden, Germany); mouse anti-p100/p52 (#05-361; Merck Millipore, Darmstadt, Germany), mouse anti-beta-actin (#A5441; Sigma-Aldrich, Steinheim, Germany); followed by horseradish peroxidase-conjugated goat anti-mouse (SC-2005), goat anti-rabbit (SC-2004) or donkey anti-goat (SC2020; Santa Cruz Biotechnology, Santa Cruz, USA) antibodies. For immunoprecipitation, cells were lysed in 10 mM Tris (pH 8.0), 150 mM NaCl and 1% Nonidet P-40, supplemented with protease inhibitor (Roche, Grenzach, Germany), and incubated (4 °C) with mouse anti-caspase-8 (as above) and pan-mouse-IgG Dynabeads (#11041; Invitrogen/Life Technologies, Darmstadt, Germany), washed, and analyzed by western blot. Cell death, cytochrome-*c* release, activated caspase-3 and mitochondrial membrane potential were assessed as previously described.^7, 9, 10, 43^ TNF-*α* was measured in cellular supernatants by a bead-based immunoassay (Fluorokine MAP cytokine, R&D Systems) and a BioPlex analyzer (Bio-Rad, Munich, Germany).

### Statistical analysis

IC_50_ were calculated based on three independent experiments performed in triplicates, different conditions obtained from three independent experiments performed in triplicates were compared by Mann-Whitney *U*-test (Prism 5.0, GraphPad, La Jolla, USA). Leukemia-free survival was analyzed by Kaplan-Meier and log-rank tests (SPSS 19.0, IBM, Munich, Germany), one recipient (VDA+BV6) moribund not due to leukemia was censored. *P*-values <0.05 were considered as significant.

## Figures and Tables

**Table 1:** Characteristics of patients and derived primograft leukemia samples

	**N**	**%**
Total	40	100
		
*Primograft derived from*		
Initial diagnosis	37	93
Relapse	3	7
		
*Gender*		
Male	23	58
Female	17	42
		
*Age (years)*		
1–9	27	68
<1 or >9	13	32
		
*Immunophenotype*		
Pro-B ALL	4	10
c-ALL	26	65
Pre-B ALL	10	25
		
*Gene alterations*		
*ETV6*/*RUNX1*	7	17
*BCR*/*ABL*	1	3
*MLL*-rearrangement	4	10
None of the above	28	70
		
*BFM high risk*		
Yes	5	13
No	32	80
Not applicable	3	7
		
*Prednisone response*		
Poor	1	3
Good	36	90
Not applicable	3	7
		
*Resistance to spontaneous apoptosis*		
Yes	5	13
No	11	27
Not analyzed	24	60
		
*Time to leukmia (TTL)*		
Short	11	27
Long	29	73

## Abbreviations

ALL: acute lymphoblastic leukemia
AML: acute myeloid leukemia
BCP-ALL: B-cell precursor acute lymphoblastic leukemia
BFM: Berlin Frankfurt Münster
BIR: baculoviral IAP repeat
cIAP: cellular inhibitor of apoptosis protein
CLL: chronic lymphoblastic leukemia
DISC: death-inducing signaling complex
DMSO: dimethyl sulfoxide
DSMZ: Deutsche Sammlung von Mikroorganismen und Zellkulturen
ETV6/RUNX1: Ets variant 6/Runt-related transcription factor
FADD: Fas-associated protein with death domain
HEPES: 4-(2-hydroxyethyl)-1-piperazineethanesulfonic acid
huCD19: human cluster of differentiation 19
I*κ*B: inhibitor of kappa B
IAP: inhibitor of apoptosis protein
IC_50_: half-maximal inhibitory concentration
i.p.: intraperitoneal
MLL/AF4: mixed lineage leukemia/ALL-1 fused gene on chromosome 4
MMP: mitochondrial membrane potential
NF-*κ*B: nuclear factor kappa B
NIK: nuclear factor kappa B-inducing kinase
NOD/SCID: non-obese diabetic/severe combined immunodeficiency
PARP: poly(ADP-ribose)-polymerase
PB: peripheral blood
PBL: peripheral blood lymphocytes
RING: really interesting new gene
RIP1: receptor-interacting serine/threonine-protein kinase 1
RNA: ribonucleic acid
SMAC: second mitochondria-derived activator of caspase
shRNA: short hairpin RNA
siRNA: small interfering RNA
T-ALL: T-cell acute lymphoblastic leukemia
TNF: tumor necrosis factor
TNF-*α*: tumor necrosis factor alpha
TNFR: tumor necrosis factor receptor
TP53: tumor protein p53
TTL: time to leukemia
TTR: time to leukemia reoccurrence
VDA: vincristine, dexamethasone, asparaginase
zVAD.fmk: *N*-benzyloxycarbonyl-Val-Ala-Asp fluoromethylketone
