# Supplementary material for: Intrinsic and chemo-sensitizing activity of SMAC-mimetics on high-risk childhood acute lymphoblastic leukemia
Source: Cell Death Dis. 2016 Jan 14;7(1):e2052–. doi: 10.1038/cddis.2015.382 (PMC4816168; doi:10.1038/cddis.2015.382)
Supplement: Supplementary Information [file cddis2015382x1.pdf]

SUPPLEMENTARY INFORMATION

Intrinsic and chemo-sensitizing activity of SMAC-mimetics on high-risk childhood acute lymphoblastic leukemia

Melanie Schirmer, Luca Trentin, Manon Queudeville, Felix Seyfried, Salih Demir, Eugen Tausch, Stephan Stilgenbauer, Sarah Mirjam Eckhoff, Lüder Hinrich Meyer and Klaus-Michael Debatin

---

1. **Supplementary Figure 1**
2. **Supplementary Figure 2**
3. **Supplementary Figure 3**
4. **Supplementary Figure 4**
5. **Supplementary Figure 5**

Supplementary Figure 1

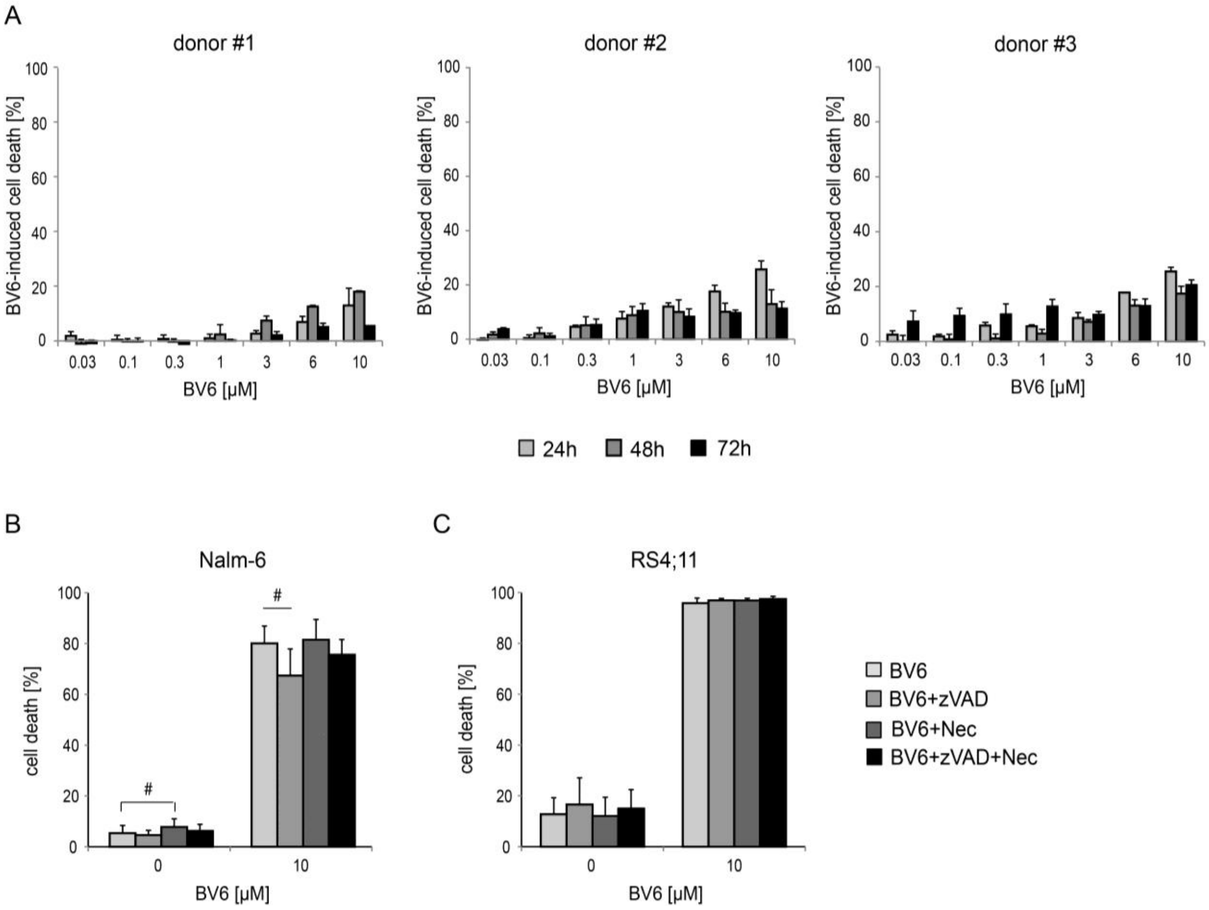

Supplementary Figure 1A: Insensitivity of healthy PBLs for BV6 induced cell death

No cell death induction in peripheral blood lymphocytes (PBLs) from healthy donors (n=3) upon BV6 exposure at low concentrations efficacious on ALL cells. Cell death by flowcytometry according to forward and side scatter criteria after treatment with BV6 at indicated concentrations after indicated time points, BV6-induced cell death was calculated as the difference of total and spontaneous cell death, mean and standard deviation of triplicate measurements are shown.

Supplementary Figure 1B-C: Absence of necroptotic cell death

Inhibition of BV6-induced cell death (48 hours exposure to BV6 at indicated concentrations)

by 20µM zVAD.fmk (zVAD), 30µM Necrostatin-1 (Nec), or the combination of zVAD.fmk and Necrostatin-1 in Nalm-6 cells (B) and in RS4;11 cells (C). Percentages of dead cells were estimated by flowcytometry according to forward and side scatter criteria, mean and standard deviation of three independent experiments each performed in triplicate are shown, comparison of BV6 to BV6 and the respective inhibitors are indicated, significance by Mann-Whitney U-test, [#] $p < .05$;

Supplementary Figure 2

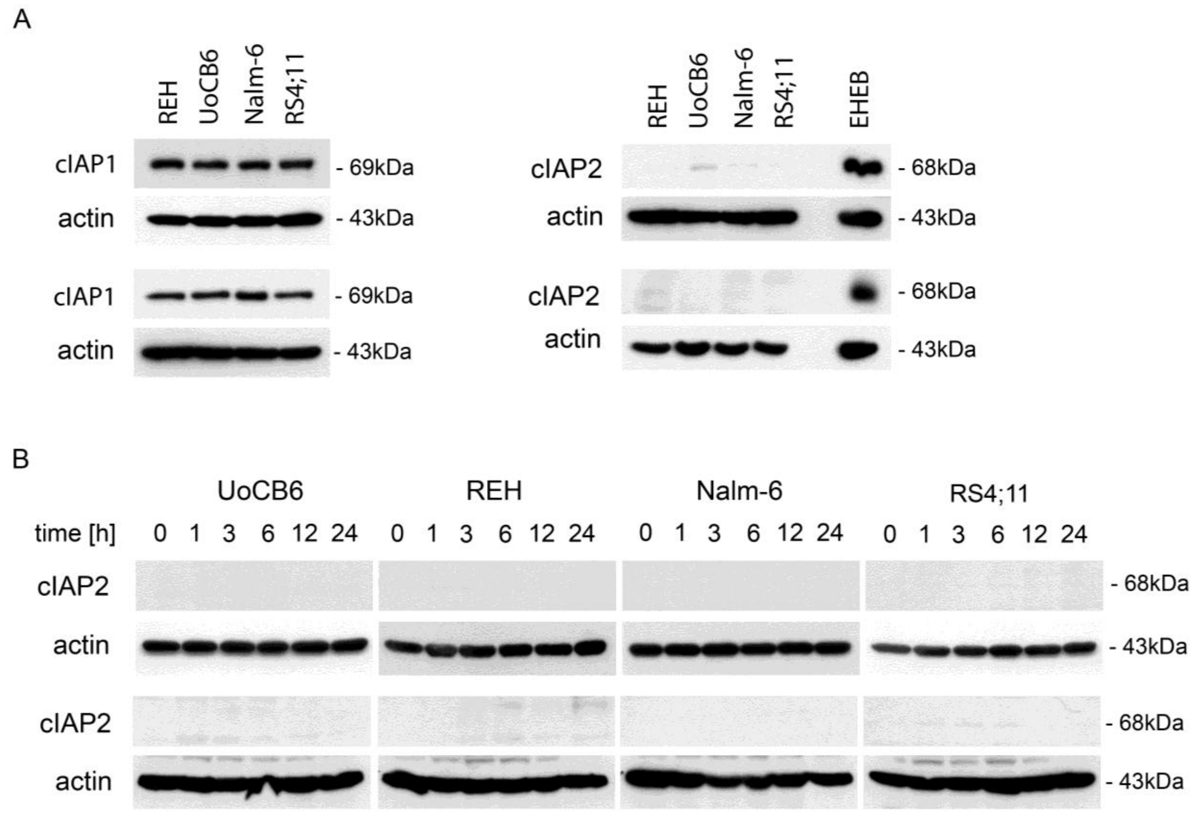

Supplementary Figure 2: No constitutive nor BV6-induced expression of cIAP2 in BCP-ALL cells

(**A**) Constitutive protein expression of cIAP1 but very low cIAP2 expression in untreated BCP-ALL cell lines. (**B**) Very low cIAP2 expression not affected upon exposure to BV6 after indicated time points (UoCB6, Reh: 1µM; Nalm-6, RS4;11: 10µM). Western Blot analysis, EHEB CLL cells were used as positive control for cIAP2, ß-actin as loading control, two replicate blots are shown.

Supplementary Figure 3

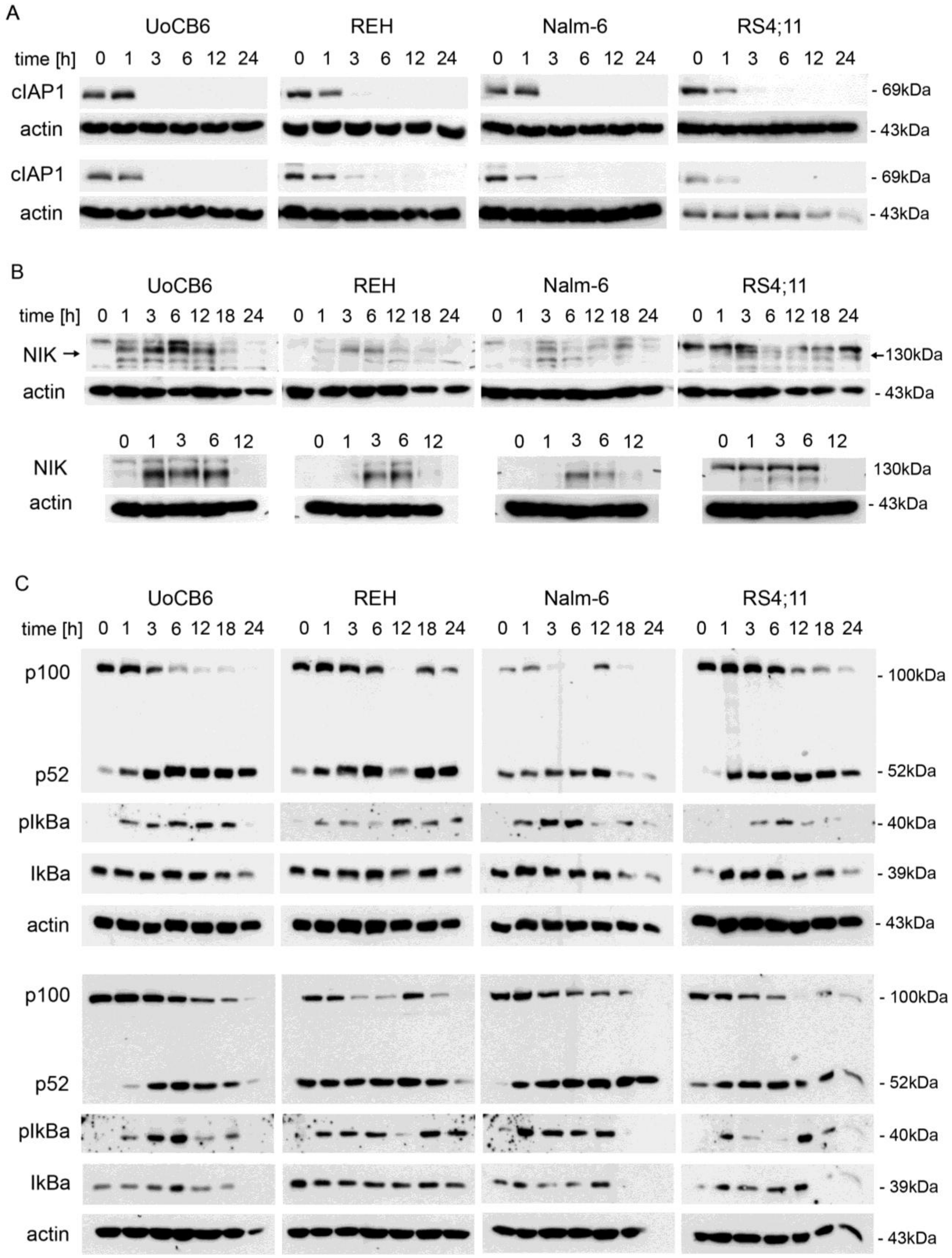

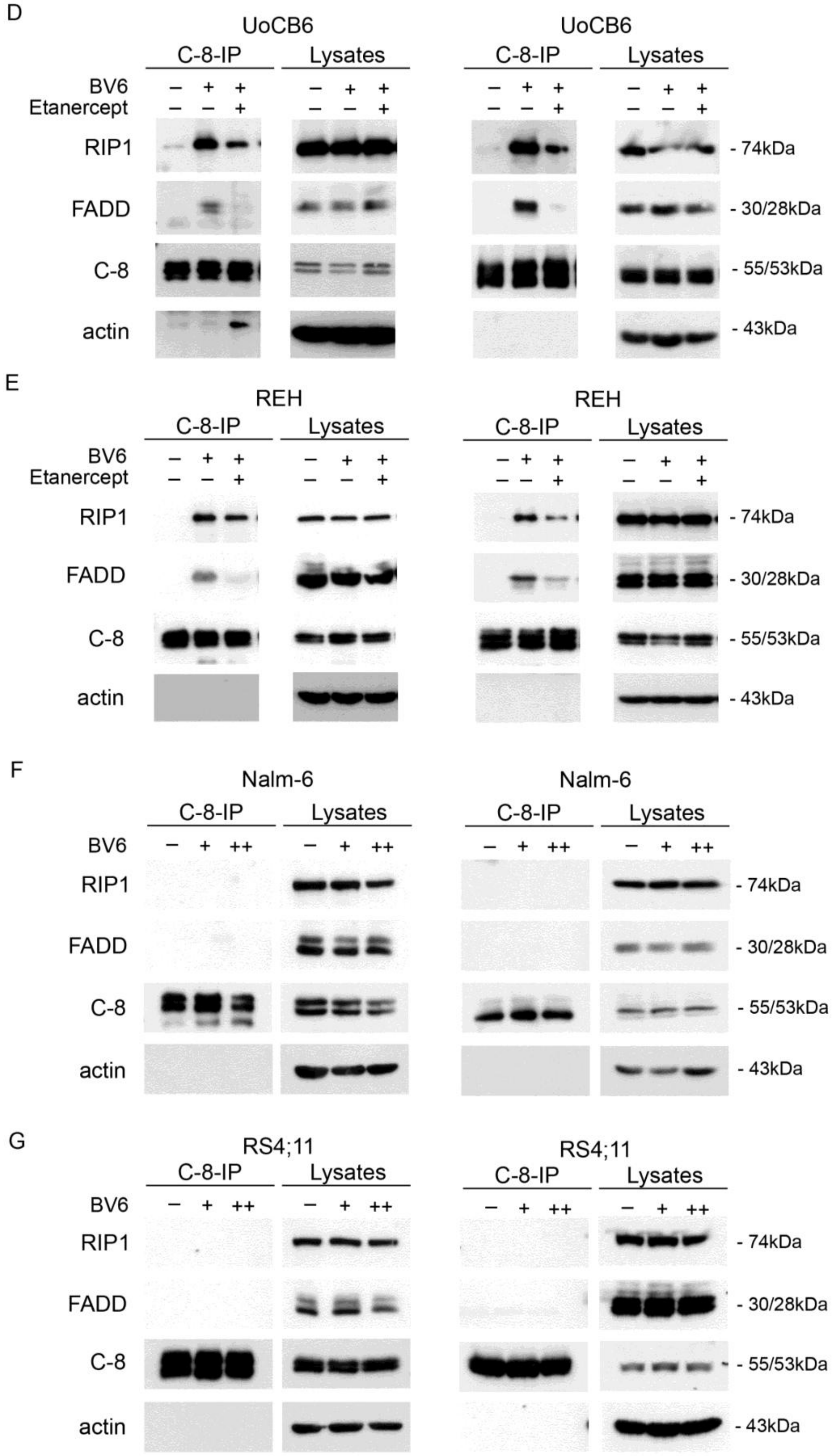
D
UoCB6
C-8-IP
Lysates
BV6
Etanercept
RIP1
FADD
C-8
actin
- 74kDa
- 30/28kDa
- 55/53kDa
- 43kDa
E
REH
C-8-IP
Lysates
BV6
Etanercept
RIP1
FADD
C-8
actin
- 74kDa
- 30/28kDa
- 55/53kDa
- 43kDa
F
Nalm-6
C-8-IP
Lysates
BV6
RIP1
FADD
C-8
actin
- 74kDa
- 30/28kDa
- 55/53kDa
- 43kDa
G
RS4;11
C-8-IP
Lysates
BV6
RIP1
FADD
C-8
actin
- 74kDa
- 30/28kDa
- 55/53kDa
- 43kDa

Supplementary Figure 3: Additional blots to Figure 2

Western Blot analysis of (**A**) cIAP1 degradation, (**B**) NIK accumulation, and (**C**) NF-κB activation in BCP-ALL cell lines exposed to BV6 for indicated time points (UoCB6, REH: 1µM BV6; Nalm-6, RS4;11: 10µM BV6; or equivalent doses of DMSO for 24 hours). For each analysis, two additional replicates are shown. Arrows indicate NIK. (**D**-**G**) TNFR complex II formation upon BV6 exposure (UoCB6 and REH: 1µM; Nalm-6 and RS4;11: 1µM [+] or 10µM [++] BV6) and dependency on TNF-α (with or without 40µg/ml Etanercept). Caspase-8 immunoprecipitation in the presence of 10µM zVAD.fmk (UoCB6, REH and Nalm-6) and Western Blot analysis of indicated proteins (two additional replicates are shown).

Supplementary Figure 4

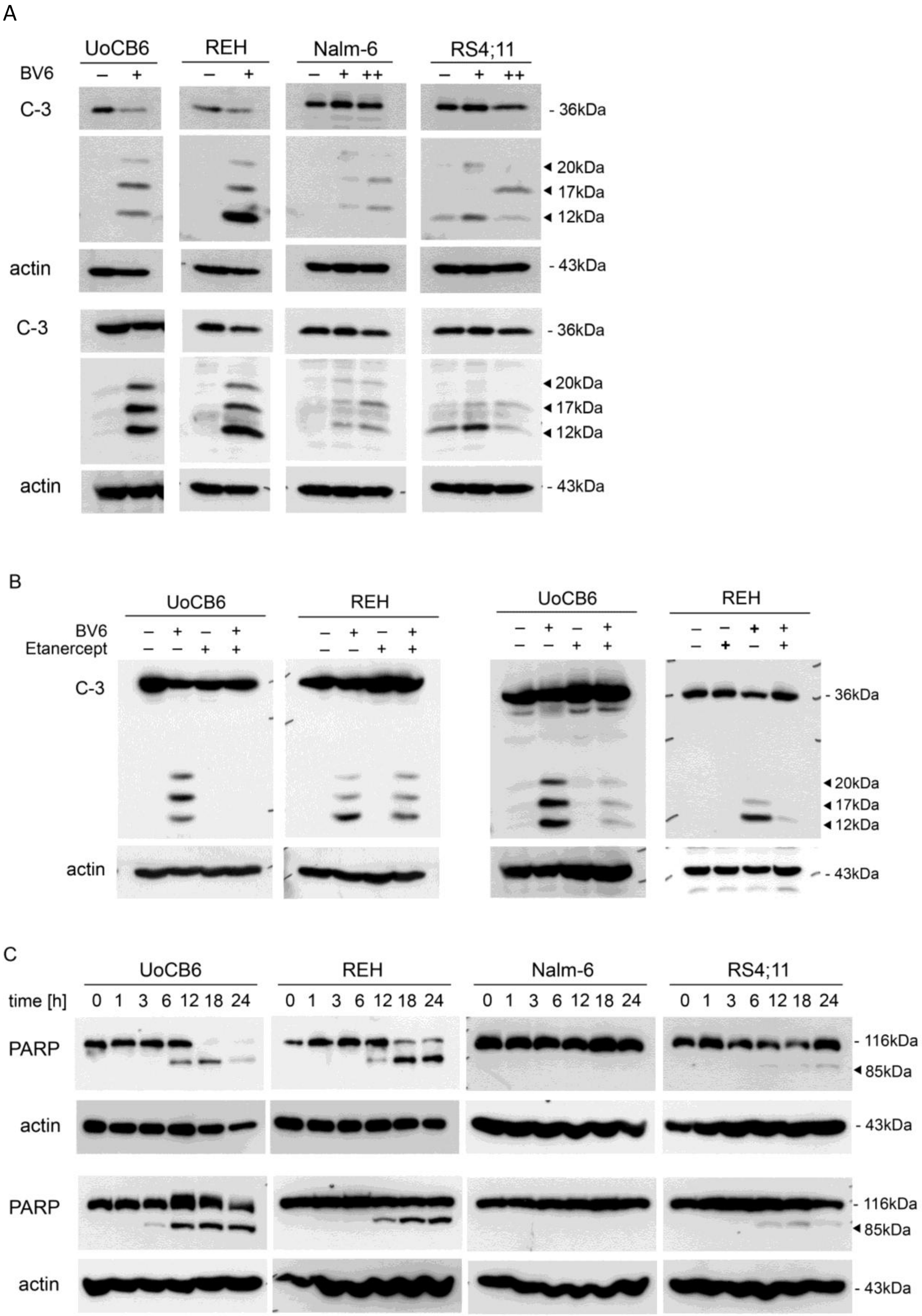

Supplementary Figure 4: Additional blots to Figure 3

Western Blot analysis of (**A**) cIAP1 degradation, (**B**) NIK accumulation, and (**C**) NF-κB activation in BCP-ALL cell lines exposed to BV6 for indicated time points (UoCB6, REH: 1µM BV6; Nalm-6, RS4;11: 10µM BV6; or equivalent doses of DMSO for 24 hours). For each analysis, two additional replicates are shown. Arrows indicate NIK. (**D**-**G**) TNFR complex II formation upon BV6 exposure (UoCB6 and REH: 1µM; Nalm-6 and RS4;11: 1µM [+] or 10µM [++] BV6) and dependency on TNF-α (with or without 40µg/ml Etanercept). Caspase-8 immunoprecipitation in the presence of 10µM zVAD.fmk (UoCB6, REH and Nalm-6) and Western Blot analysis of indicated proteins (two additional replicates are shown).

Supplementary Figure 5

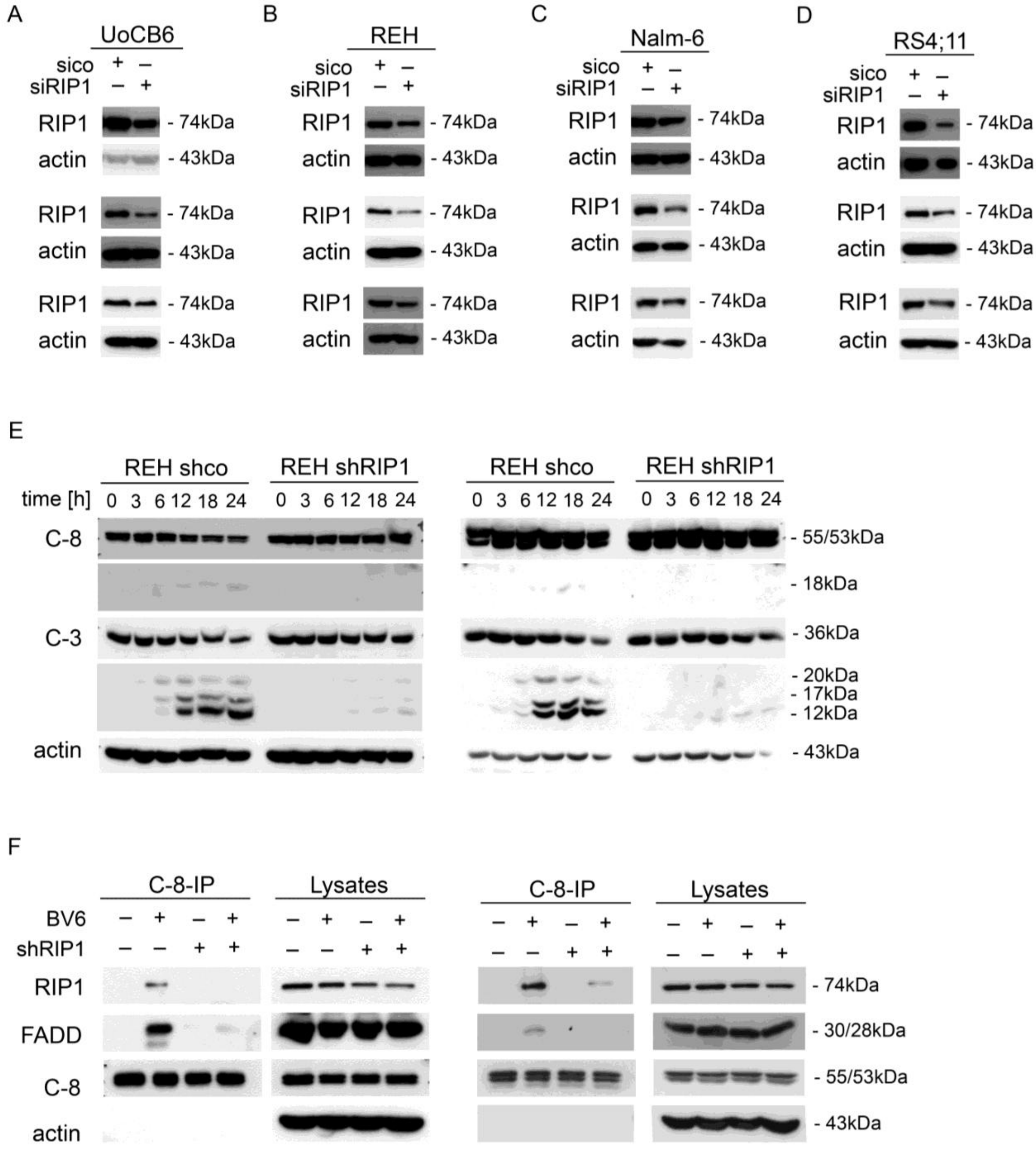

Supplementary Figure 5: Additional blots to Figure 4

(**A-D**) Efficient RNA interference mediated RIP1 knockdown (RIP1 siRNA pool, siRIP1; non-targeting control siRNA pool, sico) in BCP-ALL cells was assessed 48 hours after nucleofection by Western Blot analysis. Blots from three independent experiments are shown. (**E**) Absent caspase-8 and -3

activation (1 µM BV6 for indicated time points) in REH cells upon shRNA-mediated RIP1 knockdown, as well as (**F**) impaired complex II formation (1 µM BV6 for 18 hours, caspase-8 immunoprecipitation in the presence of 10µM zVAD.fmk and Western Blot analysis of indicated proteins). Two additional replicates are displayed.
